# New medicines for childhood-onset systemic lupus erythematosus: an EU perspective on paediatric drug development

**DOI:** 10.3389/fmed.2025.1583140

**Published:** 2025-07-10

**Authors:** Roberto De Lisa, Fernando de Andres-Trelles, Ralph Bax, Sara Galluzzo, Brian Alward, Andrew Thomson, Dominik Karres, Hermine I. Brunner, Nicolino Ruperto, Gunter F. Egger

**Affiliations:** ^1^Paediatric Medicines Office, European Medicines Agency (EMA), Amsterdam, Netherlands; ^2^Paediatric Committee (PDCO), European Medicines Agency (EMA), Amsterdam, Netherlands; ^3^Universidad Complutense de Madrid, Departamento de Farmacología y Toxicología, Madrid, Spain; ^4^Bambino Gesù Children’s Hospital, Istituto di Ricovero e Cura a Carattere Scientifico (IRCCS), Rome, Italy; ^5^Health Products Regulatory Authority, Dublin, Ireland; ^6^Methodology Workstream, European Medicines Agency (EMA), Amsterdam, Netherlands; ^7^Pediatric Rheumatology Collaborative Study Group Cincinnati Children’s Hospital Medical Center, University of Cincinnati, Cincinnati, OH, United States; ^8^Fondazione Istituto di Ricovero e Cura a Carattere Scientifico (IRCCS) San Gerardo dei Tintori, Pediatria/Paediatric Rheumatology International Trials Organisation (PRINTO), Monza, Italy; ^9^Università degli Studi di Milano-Bicocca, Dipartimento di Medicina e Chirurgia, Milan, Italy

**Keywords:** paediatric systemic lupus erythematosus, childhood systemic lupus erythematosus, paediatric extrapolation, paediatric drug development, paediatric medicine development, European Medicines Agency

## Abstract

Childhood-onset systemic lupus erythematosus (cSLE, also known as juvenile or paediatric SLE) is a severe autoimmune disease affecting multiple organs and systems, with higher morbidity and severity compared to adult SLE (aSLE). The European Union’s Paediatric Regulation No. 1901/2006 mandates the agreement of a paediatric investigation plan (PIP) for new investigational medicinal products (IMPs) to ensure reliable efficacy and safety data for paediatric indications. This study examined the experience of the Paediatric Committee (PDCO) at the European Medicines Agency (EMA) in assessing PIPs for cSLE, highlighting the challenges and potential solutions in the planning of development of novel agents in this disease and providing the academia point of view on key points of cSLE medicines development. Regulatory requirements so far have been rather consistent when a PIP is agreed, and recommend randomised controlled trials to enable a full benefit-risk assessment. However, PIPs are agreed when adult efficacy data are not yet available and as soon as the product lifecycle progresses, new methods and approaches can offer some advantages over randomised controlled trials in this setting and might provide a comparable level of evidence of efficacy in an alternative way. Extrapolation of adult efficacy data to paediatrics is one possible approach, provided that adult trials produce data that can be used for this purpose and that the degree of residual uncertainty is appropriately quantified for the medicinal product in question. The study also highlights the value of enhanced international cooperation among regulatory authorities, developers, and academic institutions in this field to support collaborative efforts among various stakeholders.

## Introduction

Childhood-onset systemic lupus erythematosus (cSLE, also known as juvenile or paediatric SLE) is considered the paediatric equivalent of adult SLE (aSLE) (1). The disease affects multiple organs and systems, with unpredictable flare-ups and high morbidity and mortality (2). There is a growing understanding of the differences in age-dependent disease manifestation and severity between cSLE and aSLE (3). Generally, cSLE continues into adulthood and, on average, 20% of adults with SLE had an onset in paediatric age (4). With the median age of cSLE onset being around 12 years, cSLE is extremely rare below 5 years of age (5). Indeed the forms of cSLE with an onset before the age of 5 are usually rare monogenic variants (such as cases resulting from mutations in the complement system genes), or classified as neonatal lupus erythematosus (autoimmune disease acquired *in utero* as a result of transplacental passage of maternal anti-Sjögren’s-syndrome-related antigen A (anti-SSA/Ro), anti-Sjögren’s-syndrome-related antigen B (anti-SSB/La), or anti-U1 ribonucleoprotein (anti-U1-RNP) antinuclear autoantibodies) (6).

Despite limited availability of data comparing cSLE and aSLE, while cSLE and aSLE are thought to share a similar pathophysiology, evidence indicates that cSLE presents a more severe disease course, both in terms of signs and symptoms and of higher disease activity at onset (7). Paediatric patients also differ with a higher male to female ratio, higher frequency of involvement of some organ systems [e.g., more frequent kidney involvement with lupus nephritis (LN) and neuropsychiatric involvement] and quicker accrual of organ damage than adults (8).

Lupus nephritis, representing up to 40% of cSLE cases in children, is the most important clinical phenotype and deserves dedicated and new effective treatment strategies (9).

Hydroxychloroquine, glucocorticoids, and immunosuppressive medicines have been the cornerstone of lupus treatment in both children and adults. It remains a topic of ongoing debate whether it is always necessary to conduct new randomised controlled clinical trials in paediatric patients to obtain a paediatric indication for a new investigational medicinal product (IMP), when adult data have already been collected in appropriately designed clinical trials (10).

The Paediatric Regulation No. 1901/2006 (11) sets up a system of requirements, rewards and incentives to ensure that medicinal products are researched, developed and authorised to meet the therapeutic needs of children in the European Union (EU). Applicants must propose a paediatric investigation plan (PIP) and/or request a waiver whenever they are developing new active substances or products previously authorised when these are under patent and concern a new indication, pharmaceutical form or route of administration.

In the EU, therapeutic options with a centralised marketing authorisation for the treatment of aSLE are belimumab (Benlysta) (12) and anifrolumab (Saphnelo) (13), whereas voclosporin (Lupkynis) (14) is indicated for the treatment of LN in adults. Conversely, to date, only belimumab has received a centralised marketing authorisation for the treatment of SLE in patients aged 5 years and older.

A PIP outlines the studies needed to ensure that efficacy and safety data are sufficiently reliable to support a paediatric indication in the EU. Waivers are provided based on legal and scientific criteria. A PIP must be agreed by the Paediatric Committee (PDCO) at the European Medicines Agency (EMA) and must be submitted by the applicant before completion of human PK/PD studies in adult patients. After PDCO agreement, a PIP can be modified, following appropriate request, if the plan shows difficulties with its implementation as to render the plan unworkable or becomes no longer appropriate. The Committee for Medicinal Products for Human Use (CHMP) “Guideline on clinical investigation of medicinal products for the treatment of systemic lupus erythematosus and lupus nephritis” was published in 2013, also covering cSLE (15). Developers can also seek scientific advice and protocol assistance by the CHMP through the recommendation of the Scientific Advice Working Party (SAWP).

Since 2007, the PDCO agreed PIPs for 22 medicinal products for treating cSLE. Despite available guidelines (15), stakeholders call for more guidance to streamline medicine development in this field (10). Due to the lower prevalence of cSLE compared to aSLE, it is challenging to conduct clinical studies that both include a number of paediatric participants sufficient to produce reliable, self-standing evidence of efficacy and that can be completed within a reasonable timeframe.

There is increasing evidence on the potential use of extrapolation of adult efficacy data to reduce clinical trial requirements supporting a cSLE indication. Such requirements should be individualised according to the mechanism of action of the medicine and the similarity of disease manifestations in children and adults (16), but other simplified methods in the design of clinical trials could also be considered (10).

This study reviews the experience of the PDCO with PIPs agreed so far for cSLE and analyses the challenges in devising a successful development plan in this area at both the planning and at the execution phase. This is performed first by identifying common trends and unresolved knowledge gaps at the beginning of a product lifecycle, when a PIP is normally submitted for agreement of the PDCO, and then proposing potential future directions, when necessary, updates to the PIP to make it workable become necessary.

## Materials and methods

We analysed key aspects of clinical trials included in initial PIPs and any modification(s) accepted by the PDCO for medicinal products intended for the treatment of cSLE. For each PIP, we gathered the following documents from the EMA database: EMA/PDCO Opinion, and the minutes of the PDCO discussions. These documents were used to collect information on the development proposed by the applicant and on the final, agreed “key elements” that detail the minimum requirements of the study protocol that must be fulfilled by the sponsor.

In particular, we collected information on trial characteristics (e.g., study design and population) as well as perspectives from the PDCO.

We created a database that was divided into four main sections: (1) basic product information, (2) clinical studies listed in the PIP, (3) planned completion date of the PIP, and (4) development status of the product. The basic product information included the PIP procedure number, the date of PIP submission and its timeliness according to the Paediatric Regulation, International Non-proprietary Name (INN), active substance, modality type, therapeutic area, target and mechanism of action of the product, intended adult indication, and intended paediatric indication. The clinical studies information included study design, study population, sample size, endpoints, and study duration.

We reviewed all PIPs submitted for the treatment of (c)SLE since entry into force of the EU Paediatric Regulation in 2007 until December 2024 and, where applicable, related EMA scientific advice procedures. Information on development proposals for each IMP was gathered and validated using data available in EMA public assessment reports, EMA/PDCO summary reports and scientific literature.

As part of the review of the (adult) development status of each IMP we conducted a search in the AdisInsight database (17) to search for information on current development status and for discontinued development in EudraCT (18).

Figures were reported with descriptive intent, and no statistical inference was planned.

## Results

The PDCO evaluated 27 individual IMP submissions for PIPs in SLE, covering substances with various mechanisms of action. Four PIP submissions were withdrawn before a final agreement could be reached, one was under evaluation at the time of this analysis, and 22 PIPs had received a positive PDCO Opinion (Table 1).

**TABLE 1:** List of PIP submitted for PDCO agreement for SLE until 2024.

PIP number	PIP IMP	Mechanism of action	Pediatric development and PlP status	Adult development status in SLE/LN^1^
EMEA-000118-PIP03-15	Abatacept	T cell activation inhibitors	PIP agreed (discontinued)	Discontinued
Not assigned	Not available	Anti-CD20 monoclonal antibodies	Withdrawn	Discontinued
EMEA-000311-PIP06-18	Ustekinumab (Stelara)	Interleukin 12 inhibitors; Interleukin 23 inhibitors	PIP agreed	Phase III (59)
EMEA-000380-PIP06-19	Secukinumab (Cosentyx)	IL17A protein inhibitors	PIP agreed (discontinued)	Discontinued
EMEA-000520-PIP02-13 EMEA-000520-PIP01-08	Belimumab	B cell activating factor inhibitors	PIP agreed	Adult EU indication granted
EMEA-000802-PIP02-11	Tabalumab	B cell activating factor inhibitors	PIP agreed (discontinued)	Discontinued
EMEA-001207-PIP02-19	Obinutuzumab	Anti-CD20 monoclonal antibody	PIP agreed	Phase III (60)
EMEA-001220-PIP05-19	Baricitinib	Janus kinase (JAK) inhibitor	PIP agreed (discontinued)	Discontinued
EMEA-001295-PIP01-12	Epratuzumab	Anti-CD22 monoclonal antibody	PIP agreed (discontinued)	Discontinued
EMEA-001435-PIP02-16	Anifrolumab (Saphnelo)	Interferon alpha beta receptor antagonists	PIP agreed	Adult EU indication granted
Not assigned	Not available	Interferon alpha inhibitors	Withdrawn	Discontinued
Not assigned	Not available	Anti-TWEAK [tumour necrosis factor (TNF)-related weak inducer of apoptosis] monoclonal antibody	Withdrawn	Discontinued
EMEA-001741-PIP09-23	Upadacitinib (Rinvoq)	Janus kinase (JAK) inhibitor	PIP agreed	Phase III
EMEA-001972-PIP01-16	Blisibimod	B cell activating factor inhibitors	Withdrawn	Discontinued
EMEA-002004-PIP01-16	Atacicept	B cell activating factor inhibitors	PIP agreed	Phase III
EMEA-002264-PIP01-17	Voclosporin (Lupkinys)	Calcineurin inhibitor	PIP agreed	Adult EU indication granted (LN)
EMEA-002284-PIP02-19	Evobrutinib	Bruton tyrosine kinase (BTK) inhibitor	Under evaluation (clock-stop)	N/A
EMEA-002338-PIP03-21	Ianalumab	B-cell activation factor receptor antagonists	PIP agreed	Phase III
EMEA-002350-PIP03-20	Deucravacitinib	Inhibitor of tyrosine kinase 2 (Tyk2)	PIP agreed	Phase II/III
EMEA-002374-PIP01-18	Avizakimab	Anti-interleukin 21 antibody	PIP agreed	Phase I/II
EMEA-002555-PIP02-21	Litifilimab	Anti-blood dendritic cell antigen 2 (BDCA2) antibody	PIP agreed	Phase III
EMEA-002702-PIP01-19	Dapirolizumab pegol	Antibody fragment against the CD40 ligand (CD40L)	PIP agreed	Phase III
EMEA-002815-PIP01-20	Rozibafusp alfa	B cell activating factor inhibitors	PIP agreed	Phase II
EMEA-002824-PIP01-20	Telitacicept	B cell activating factor inhibitors	PIP agreed	Phase II
EMEA-003108-PIP01-21	Cenerimod	Sphingosine-1-phosphate receptor 1 (S1P1) modulator	PIP agreed	Phase III
EMEA-003156-PIP01-21	Efavaleukin alfa	Regulatory T-lymphocyte stimulants	PIP agreed (discontinued)	Discontinued
EMEA-003342-PIP02-22	Enpatoran	Toll-like receptor 7/8 antagonists	PIP agreed	Phase II

An application for PIP can be withdrawn before the PDCO agrees on a final Opinion. Pre-opinion withdrawal of a PIP could occur, for instance, once adult (negative) results become available during the timespan required to agree a PIP (a 120 day procedure, with a clock-stop). After an Opinion has been agreed the PIP cannot be withdrawn any longer but discontinuation following appropriate communication with the EMA can be notified. The PIP number is a unique number assigned by EMA for each procedure. Using such number, it is possible to find the corresponding EMA Decision, which lists of the studies of the PIP, on the EMA website. Public assessment reports related to the product and its Product Information, if the product is already authorised, are also available.

^1^Data collected from AdisInsights (14).

There were seven PIPs for existing medicinal products that had an existing indication for the treatment of another adult disease other than SLE. For the remaining PIPs, the products were not authorised in the EU at the time of the PIP agreement.

For all PIPs, a waiver for the paediatric population below 5 years of age was agreed by the PDCO and studies in children under 5 were therefore not included in any PIP. This approach is consistent with the relevant CHMP guideline that states that there is no need for development of medicines for SLE in children under 5 years of age as the disease is extremely rare in this age group.

### Study design

**FIGURE 1:**
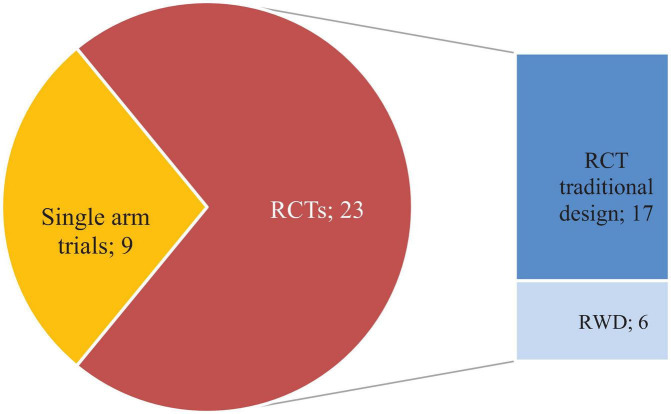
Trials included in all PIPs agreed for paediatric SLE. Each PIP agreed by the PDCO lists all the required clinical studies to be performed to obtain a marketing authorisation in the paediatric population provided results show a positive benefit risk balance. Every PIP can include one or more studies. This figure shows the types of study design included in all PIPs agreed so far: Randomised Controlled Trials (RCT) and Randomised Withdrawal Design Trials (RWD). The clinical studies considered cover both studies supporting an indication in paediatric SLE and those specifically targeting LN. Agreed RCTs were either parallel design versus active or placebo or withdrawal design versus active or placebo.

Fourteen out of the 22 accepted PIPs comprised one single planned clinical study to address pharmacokinetics (PK), safety and efficacy, 7 PIPs included two studies of which one was focused on PK and the second one on safety and efficacy, and 1 PIP consisted of four studies. The study design of trials included in the PIPs is shown in Figure 1. All PIPs for SLE featured at least one double-blind randomised controlled trial (RCT) comparing add-on use of an IMP with add-on placebo. Three PIPs, restricted to treatment of LN, included solely single-arm trials, but these intend to collect RCT evidence in adolescents aged 12–18 as part of the same PIP.

### Study population

Childhood-onset systemic lupus erythematosus is classified using the same criteria used for aSLE. This is considered justified, given the similarities in disease characteristics between children and adults (19, 20).

In alignment with the planned adult indication, 22 PIPs focused on patients with cSLE, without any requirements for including specific disease subpopulations. Five PIPs primarily targeted patients with LN.

The main inclusion criteria were diagnosed SLE, classified according to the European Alliance of Associations for Rheumatology (EULAR)/ACR criteria (21) and age of onset before 18 years. Patients were classified based on a positive antinuclear antibody (ANA) test at an adequate titre, with criteria grouped into seven clinical domains (constitutional, haematologic, neuropsychiatric, mucocutaneous, serosal, musculoskeletal, and renal) and three immunological domains (antiphospholipid antibodies, complement proteins, and SLE-specific antibodies), weighted from 2 to 10.

In addition, in almost all (19 out of 22) PIPs, the key elements of the PDCO opinion stipulated that studies included only patients with moderate or severe disease activity on a standard background medication (e.g., oral glucocorticoids and hydroxychloroquine) and/or patients who were intolerant or insufficiently controlled on first-line steroid-sparing immunosuppressors (e.g., methotrexate, azathioprine, and mycophenolate mofetil). In all PIPs, studies required that patients had disease activity ≥6 on the SLE Disease Activity Index 2000 (SLEDAI-2K), as well as clinical manifestations of active disease (22).

The PDCO explicitly requested that patients with known LN and/or central nervous system (CNS) manifestation on stable maintenance therapy can also be included in the planned studies of six PIPs that were not specifically targeting the treatment of active LN, if they meet other inclusion criteria.

### Sample size

The average number of patients planned to be recruited in the main study of each PIP was 78 (median = 70, min = 52, max = 160), across both paediatric age groups from 5 to less than 12 and from 12 to less than 18 years of age. For PIPs targeting LN, the average number of patients originally requested to be recruited by the PDCO was at least 40 (average = 48, median = 40). As pre-pubertal onset of SLE is uncommon, there was a reduced number of patients in each PIP for the subgroup of patients aged between 5 to less than 12 years (average = 12, min = 10, max = 20).

In general, sample sizes were initially established based on preliminary evidence to show with 80%–90% power a true response rate for the selected primary endpoint in a purely frequentist framework for an independent trial, without the incorporation of any prior information/degree of belief. However, updates on sample sizes requested in the PIP were generally expected once more accurate models became available upon the analysis of adult data.

### Endpoints

In seven PIPs, the primary endpoint was the *proportion of participants who achieve at least a Responder Index (SRI)-4* (23) response at the end of study period. In six PIPs, it was *time to or number of flares for the observed period*. Other primary endpoints were the *point reduction from baseline in* SLEDAI-2K (two PIPs) and *BILAG-based composite lupus assessment* (BICLA) at the end of study period (two PIPs). In all five PIPs for products targeting LN, the primary endpoint was *complete renal response* (CRR).

In line with the CHMP guideline, achievement of *responder status/defined improvement according to Paediatric Rheumatology InterNational Trials Organisation/American College of Rheumatology juvenile SLE response PRINTO-ACR* (12) was also requested to be evaluated as a secondary endpoint for 15 PIPs.

All clinical studies had PK and/or pharmacodynamic (PD) endpoints to confirm the paediatric dose selected. In 19 of the 22 agreed PIPs, one or more modelling and simulation analyses were explicitly requested and described; these consisted of population pharmacokinetic (PopPK) and potentially also pharmacokinetic/pharmacodynamic (PopPK/PD) models.

### Study duration

Analysis of all PIPs showed that, for all RCTs included, treatment duration for the evaluation of the primary endpoint was at least 48 weeks.

### Timelines

**FIGURE 2:**
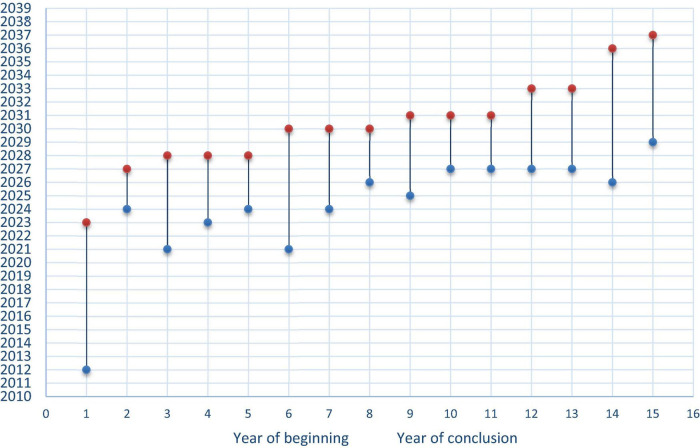
Planned duration of agreed PIPs. In this graph the *X*-axis reports each PIP and its expected start and completion date, expressed in years, and reported in the *Y*-axis. This is based on the agreed start and completion dates reported in the PDCO Opinion. It is sometimes possible that such completion date in the planning phase does not match the real completion date since it is only a projected one. Some developments might take longer than expected and amendment of the dates agreed in the PIP can be granted to sponsors upon justified requests for modification of the agreed PIP.

The planned duration and time of completion for each PIP are shown in Figure 2 (discontinued PIPs are excluded; planned duration of each agreed PIP: the *X* axis reports each PIP in order of expected completion date – year in the *Y* axis - according to the agreed PIP. In one case the initiation date of the planned clinical studies was not known).

All PIPs were deferred, meaning results of PIP studies did not have to be submitted at the time of application for an initial marketing authorisation for adults.

So far, only one PIP has been successfully completed. The evidence collected supported an extension of indication for treatment of cSLE in the EU for belimumab.

### Discontinued or withdrawn PIPs

Five agreed PIPs were discontinued and four were withdrawn before the PDCO agreed on a final PIP. Discontinuation of these PIPs was primarily due to discontinuation of the clinical trial program when adult Phase II or Phase III with active SLE or active LN failed (Table 1).

## Discussion

To date, EMA’s PDCO has agreed 22 PIPs, covering different molecules with various mechanisms of action, including active substances targeting B-cell function, B/T-cell communication, toll-like receptors (TLRs) or interferons, or directed towards intracellular machinery. This variety of therapeutic targets reflects the wide range of novel therapies currently in clinical development (24) to address the unmet medical need in patients with SLE. There are other molecules that are currently being tested in Phase I trials for which proposals for PIPs have not yet been submitted to the EMA/PDCO (24).

Trials proposed in PIPs had been deferred, this means that they would start only when adult trials show treatment benefits and manageable product safety and a marketing authorisation application for adults has been submitted. This is also done to avoid any delay in marketing authorisation application of new IMPs and access to a new therapy by adults. While the PDCO routinely evaluates the hypothetical risk of off-label use should a product be approved for adults, it thus appears prudent to wait for the results from adult trials before enrolling paediatric patients in studies. Such proof of product safety and efficacy reduces the risk of unknown adverse reactions in children and adolescents, keeps them from being exposed to inefficacious IMPs and helps establish a medicine dose suitable for paediatric patients.

Currently, the study design required in the various PIPs is mainly RCT versus placebo as add-on to standard of care treatment. The use of placebo in paediatric trials has been considered acceptable when there is genuine equipoise between the active treatment and placebo (25). The issue of informed consent is particularly challenging with children. Generally, physicians and parents have to decide at which age or cognitive developmental stage a child can assent to study participation. Importantly, study-related information must be designed in a written or verbal format to ensure comprehension of the study activities, risks and benefits at an age appropriate level, a prerequisite to provide full assent (26).

However, paediatric trials exclude children with cSLE who adequately respond to standard of care treatment, and only focus on patients who have had an inadequate response to standard of care treatments. This ensures the best standard of care is provided and minimises the patient burden of paediatric trials with placebo control groups. However, the RCT versus placebo design in this setting has not been exempt from criticism: to demonstrate a statistically significant difference between the treatment arm and placebo, an adequate sample size is required. According to developers, recruiting the number of patients needed might prove particularly challenging, especially in children between 5 and 12 years of age. Recruitment challenges prolong study duration and potentially delay in medicine approvals. Furthermore, given the low acceptance of a placebo-controlled trial by patients, families or caregivers and treating physicians (due to concerns about subjecting a child with active cSLE to an inactive treatment), the feasibility of such a trial is a legitimate concern (10).

However, the PDCO does not consider trials with an active comparator appropriate, since no single treatment can be considered the *gold standard* in an already pre-treated, multi-refractory study population. Patients with active disease often are on different treatment regimes and both their families and physicians are unlikely to accept re-exposure to previously ineffective medicines due to the low chance of additional benefit. Lastly, novel compounds are rarely compared with active treatments in adult trials. Thus, any deviation in trial design in the paediatric population (e.g., IMP versus placebo in adults, and IMP versus active comparator in paediatrics) would hamper extrapolation of adult efficacy data and limit context and comparison in the evaluation of paediatric trial outcomes.

For some PIPs, the PDCO agreed on trials utilising a randomised withdrawal design (RWD). RWDs only assess efficacy in an enriched population, as patients who did not respond to treatment during the open-label period are discontinued from the study and responders are then randomly continued on IMP or placebo in a double-blind fashion, and efficacy is assessed by evaluating the rate of flare.

Randomised withdrawal design, mainly utilised in juvenile idiopathic arthritis (JIA), give some preliminary but important information on the potential time to drug discontinuation since many medicinal products have shown a prolonged biologic effect which extended far beyond the half-life of the product (e.g., median time to flare in the placebo group was 236 days in the canakinumab trial in systemic JIA and 95.6 weeks in the golimumab trial in JIA with polyarticular course) (27, 28). At the same time, carry-over effects may reduce the likelihood of flaring in patients switched from placebo.

Notably, the burden of active disease is likely considerably lower in the RWD design as only responders are randomised. Limiting the burden of active disease in cSLE is important as this is a major risk factor for damage acquisition, hence mortality (29, 30). In a RWD, the endpoint of clinically significant flare is similar to a no more than moderate flare of disease. However, there is no evidence from the medical literature that moderate disease worsening for a short period of time is a risk factor of damage acquisition (as reintroduction of active treatment would be expected to recapture improvement observed in the open-label part of a RWD preceding randomisation). Arguably, there is initial data from adults with SLE that flare events might promote damage acquisition (31). However, it is currently unclear whether the burden of overall disease or individual flare events constitute the primary driver of damage. Finally, an additional limitation is that results would be difficult to compare to adult data where RWD trials have not been performed.

It has been pointed out that single arm pharmacokinetic pharmacodynamic studies are preferred by some paediatric rheumatologists over double-blind parallel designs for testing IMPS in cSLE trials (10) in terms of feasibility, because they are considered more acceptable by patients and investigators facilitating recruitment.

Research participants might want to avoid the possibility to be assigned to the placebo arm even if standard of care therapy is still provided, based on the belief that once efficacy is proven in adults it is unlikely that lack of efficacy will be shown in younger patients, as long as exposure matching of the IMP is established. A study performed by the PRINTO/Pediatric Rheumatology Collaborative Study Group (PRCSG) networks showing the results of a surveys regarding future medications’ clinical trials in cSLE supports this (10). The majority of survey respondents preferred an open-label PK/PD study while the rest favoured a blinded, parallel, placebo-controlled study. This was mainly based to on the known similarities between aSLE and cSLE disease manifestations which should lead to a simplified approach essentially centred on establishing the correct dose for the same indications in adults, along with the confirmation of the safety profile in the format of post-marketing surveillance studies. Notably, most cSLE patients are 12 years or older at the time of diagnosis; hence the anticipated PK/PD is expected to be comparable to that in adults based on a large body of prior studies of IMPs. Arguably, from a safety point of view, comparative designs seem still warranted. Most IMPs for which a PIP has been agreed lack any prior data on exposure in paediatric patients as there often is no information from use of these IMPs in another indication or from authorised products in the same therapeutic class.

It is acknowledged that, so far, treatment recommendations for established therapies continue to be similar for aSLE and cSLE, but some specific considerations apply. A recent study conducted using DARWIN-EU (32) evaluated treatment patterns in adult versus paediatric patients across four European countries, showing high glucocorticoid prescriptions in paediatric patients and more intensive use of therapy in cSLE. A similar study conducted in the United States had consistent findings (33), as did a study conducted globally by the PRINTO group (8). This shows there is an existing medical need for viable, steroid-sparing, treatment alternatives paediatric patients. Importantly, such observations could also suggest that dose selection for new IMPs needs careful consideration as to whether paediatric patients may require higher IMP exposure, compared to adults, to achieve comparable therapeutic effects in terms of disease control or remission (8).

In addition to broadening understanding of the effective dose of an IMP for paediatric use, there is a critical need to obtain comprehensive data on the impact of an IMP on the (maturation of the) immune system and the clinical progression, including damage accumulation, of cSLE. These data should be gathered in a sufficiently high number of paediatric patients over an extended period of time, in order to address current information gaps. Still, safety information gathered in such clinical trials, given a relatively small sample size, would need to be complemented by post-marketing surveillance studies in a larger cSLE population, using instruments and technology now available to measure disease activity and damage for both adults and children (34).

The *Guideline on clinical investigation of medicinal products for the treatment of systemic lupus erythematosus and lupus nephritis* in fact, highlights the need for using instruments that capture disease course and the potential need for more aggressive therapy and its impact in the treatment of paediatric patients who are still growing and developing.

From a regulatory perspective, an initial PIP should include RCTs to establish the benefit-risk profile of an IMP for its first paediatric use. Once additional data from larger adult studies of the safety and efficacy of the IMP become available, proposals for alternative study designs might become acceptable. It is important to underline that currently agreed paediatric studies use specific tools that are particularly relevant to capture benefits in the paediatric population. This will add essential information supporting future licensing.

To support consideration of extrapolating adult efficacy data to children ≥5 years with cSLE, a recent study has compared efficacy and exposure-adjusted response of medicines for aSLE and cSLE. Some data were too limited to quantitatively assess the exposure–response relationship. However, results supported leveraging data from aSLE studies to inform paediatric medicine development programmes and pinpoint areas of remaining uncertainty about the risk-benefit profile of a medicine in children (16). Such uncertainties then need to be addressed in appropriately designed paediatric studies.

The increased use of modelling and simulation analyses in paediatric medicine development has greatly simplified dose finding in cSLE. However, under- or overexposure remains a risk in paediatric patients, particularly for IMPs lacking paediatric PK/PD data in other conditions or those for which such data are poorly characterised in adults. Such risk differs according to the mechanism of action and therapeutic window and, therefore, data supporting a specific extrapolation concept must be presented, discussed or planned to be generated within a PIP.

Recently, the International Conference of Harmonisation (ICH) published its E11A Guideline on paediatric extrapolation (35), providing a framework for the use of extrapolation as a tool to support paediatric medicine development. The guideline underlines the importance of identifying study designs which can address the remaining knowledge gaps and uncertainties based on an assessment of the existing data. EMA has published on its website a structured guidance on the use of extrapolation, which is a specific tool to be used when submitting a PIP that will guide applicants in presenting their extrapolation concept and plan (36). This structured guidance informs developers which data are useful and need to be collected in adults to support discussions with the PDCO on the development of an appropriately designed PIP that ensures all essential paediatric data are collected.

Additional data from paediatric trials in products belonging to the same pharmacological class might also provide support for overlapping exposure-response profiles between aSLE and cSLE for an IMP. However, to date, the PDCO has not seen a sufficiently successful precedent of pharmacological class data informing extrapolation of adult data to determine a paediatric exposure-response profile of a new IMP.

Additional tools might improve paediatric trial feasibility by using existing data from other sources (37). Such sources could include adult treatment effect data used in an extrapolation exercise or external control arm data from either adult data or other relevant RCTs. The methods used to generate such data can be either frequentist or Bayesian, as outlined in ICH E11A.

In this light, the case of belimumab is particularly relevant. At the time of EMA’s evaluation of the marketing authorisation application for the paediatric indication, the FDA requested a reanalysis of the primary endpoint using an informative Bayesian method. Conversely, the EMA acknowledged the rarity of cSLE and the challenges in recruiting paediatric patients for a Phase III study (38). The treatment effect observed in the paediatric trial resembled that in adult studies, providing sufficient evidence for licensure in patients aged 5 years and above without needing further Bayesian analyses. However, regulatory agencies can only accept these methods for paediatric extrapolation if the model chosen and its properties are well described, and it is likely to be sufficiently robust to deviations from assumptions (39).

Platform and basket study designs also appear to be viable options. Engagement between developers, global networks and the rheumatology research community is crucial for these innovative approaches to become reality. EMA’s SAWP supports innovative development methods and provides, upon request from developers, opinions on the qualification of such methods and *Letters of support* for novel methodologies that have been shown to be promising in the context of research and development into pharmaceuticals. These have included registries and platform studies (40).

The next decade will see the conclusion of several adult studies in SLE. Based on their results, new paediatric studies will be initiated, and it can be anticipated that recruitment will be competitive. Considering the PIPs agreed, most of the paediatric studies are likely to be initiated between 2025 and 2030, highlighting how competitive recruitment might become a major issue given the limited pool of potentially recruitable paediatric participants. Further, prolonged study start-up procedures in paediatric compared to adult studies are likely and need to be considered to meet anticipated study completion timelines. Decreasing development timelines seems to be the next challenge, particularly for those IMPs belonging to a class of medicines with proven efficacy in aSLE. It is important for developers to take this into account when planning a paediatric development programme, identifying suitable investigative centres early and selecting the most appropriate population to produce results matching adult data. Early submission of the PIP helps prevent delays, allowing for timely adaptation based on emerging evidence.

In this context, aligning the requirements of regulatory bodies globally regarding the necessary data for the potential approval of an IMP in a cSLE indication would facilitate consistent requirements and enable global recruitment of paediatric participants.

The European Medicines Agency regularly meets with non-EU regulators in so-called “clusters.” The clusters are areas of cooperation focussing on special topics and therapeutic areas identified as requiring an intensified exchange of information and collaboration. Paediatric study plans for the treatment of cSLE have been discussed between regulators at the paediatric cluster (41) on various occasions. In 2024, a dedicated session on study approaches for cSLE took place. These exchanges provide valuable opportunities to enhance alignment on key aspects of timelines and study requirements despite different legal processes for paediatric study plan submissions in different regions.

In contrast to adults, LN frequently appears as an initial feature of cSLE.

In a large prospective convenience cohort of patients enrolled by the PRINTO group, about 40% of patients had kidney involvement, half of them being new onset patients with active kidney disease (9). In 90% of these patients, renal disease develops within 2 years of the onset of cSLE (42). In addition, compared to aSLE, cSLE causes more frequently CNS disorders, especially seizures. Following feedback from stakeholders, the PDCO has often insisted that patients with kidney and/or CNS involvement are allowed to participate to research to address the important unmet need for these patients. In this context, it is important to address the research gap of defining appropriate inclusion criteria to ensure that potential placebo exposure in trial participants limits their risk of progression of kidney or CNS disease, as both these cSLE features are recognised as main risk factors of poor patient outcomes.

For studies targeting LN, the PDCO recognises the limitations in feasibility associated with the use of placebo in patients who have an increased risk of harm (more severe disease) when they are given non-effective treatment (43) and the limits in producing informative results of a controlled study given the reduced sample size. Due to the risk of prolonged uncontrolled disease and proteinuria, which can lead to a poor prognosis, exposing children with high renal activity to placebo in paediatric LN studies is problematic when treatment that has been demonstrated effective in adults (and can therefore be assumed effective in children) is available. Therefore, a single-arm trial paired with appropriate PK/PD analyses based on renal function parameters or on the PRINTO criteria for cSLE improvement has been considered justified so far (23).

## The academia point of view

The PRINTO had among its main line of research several projects dedicated to cSLE. In particular, the work started with a prospective data collection in 557 patients (39% of them with renal involvement). This cohort led to the development and validation of a core set of five cSLE disease severity measures (1. physician’s global assessment of disease activity, 2. global disease activity measure, 3. 24-h proteinuria, 4, parent’s global assessment of the patient’s overall wellbeing, and 5. health-related quality of life assessment) and a related definition of improvement, the so called PRINTO/ACR criteria, which required at least 50% improvement from baseline in any two of the five core set measures, with no more than one of the remaining worsening by more than 30% (23, 44). Other relevant ACR-endorsed outcome measures for cSLE are those for the measurement of minimally important improvement and clinically relevant worsening and clinical inactive disease (45, 46). These outcome measures were rigorously developed using data from over 200 children with cSLE in studies led by the PRCSG in collaboration with PRINTO. Further, the SLE Responder Index (SRI-4) and BICLA have been validated for use in cSLE as have adult response criteria utilising changes in haematuria, proteinuria and kidney function (GFR) for the assessment of LN (47). The work went further to evaluate the related therapeutic approaches, the performance in the subgroup of children with renal involvement and the effect of the disease on growth and puberty (8, 48). This initial work was thought as the essential methodological basis for the subsequent implementation of clinical trials in cSLE, including those in children with kidney disease that arguably represents the most important organ involvement with cSLE.

The PRINTO network, now grouping 95 countries and more than 700 centres, has been working with PRCSG in a North American network with over 90 sites. Hence, supported by the size of the networks and by prior site assessments (46). PRINTO and PRCSG proposed and agreed with several pharmaceutical companies that the same outcome measures used in adults should be applied also in children to facilitate comparison and extrapolation from studies in adult with SLE.

In the belimumab trial, the use of the adult criterion (the SRI4) was not able to discriminate between placebo and belimumab (49, 50). Interestingly, the PRINTO/ACR 30 and 50 criteria, used as secondary outcomes, were, on the contrary, able to discriminate between belimumab and placebo, raising the issue if criteria developed specifically for children with SLE are more suitable to depict a change between placebo and experimental therapy.

In addition, the belimumab trial enrolled 93 patients of whom only 13 (14%) were between 5 and 12 years of age and their enrolment delayed the completion of the study due to the known low prevalence in pre-pubertal children, making up about 10% of the entire cSLE population.

Inclusion of patients with active LN and/or CNS manifestation on stable maintenance therapy was recommended in some of the planned studies. This requirement should be preferably considered if the adult trials will show efficacy and/or are also targeting patients with these types of organ system involvement. Arguably, aSLE and cSLE both feature multiorgan involvement, albeit there are differences in their prevalence. Hence data from adults would assist in establishing appropriate dosing regimens in cSLE. This seems especially relevant for CNS involvement, as the brain is considered less accessible to medication given specialised blood and CSF barrier functions. Likewise, the presence of proteinuria influences medication PK, a relevant observation already shown in prior adult studies of LN (51, 52). While controlled CNS and LN involvement should not be a barrier to the enrolment to cSLE studies, specialised dosing needs for active CNS and active LN must be considered. Depending on the mechanism of action of an IMP, this could potentially raise safety concerns.

The inclusion of adolescents into aSLE studies would be another opportunity to promote the collection of data relevant to the approval of a medication in cSLE. However, this would require that adolescents with cSLE are recruited from additional study sites than those used for adult patient recruitment, would risk exposure of children to IMPs that are ultimately shown not to be effective in adults with SLE and allow for relatively high placebo exposure. This is of special concern given the known accelerated damage acquisition in cSLE compared to aSLE.

In addition, to increase feasibility of trials in cSLE, novel trial designs, which have gained momentum particularly in oncology, may be an excellent alternative to classic controlled studies (53). Such novel designs, like basket, umbrella, or platform trials, would allow for limitation of placebo exposure and newly create the option of active comparator analyses, meaning evaluation of relative effectiveness of medications.

A basket trial essentially has a master protocol study designed to test a single investigational drug or drug combination in different populations defined by disease stage, histology, number of prior therapies, genetic or other biomarkers, or demographic characteristics. An example in paediatric and adult rheumatology has been the canakinumab trial for the treatment of several recurrent autoinflammatory recurrent fever syndromes.

Umbrella trials test how well new medicines, or other substances work in patients who have the same type of disease (e.g., cancer) but different gene mutations (changes) or biomarkers. Patients receive treatment based on the specific mutation or biomarker found in their disease (54). Unfortunately, this kind of design is not yet suitable in rheumatology due to the lack of biomarkers to stratify patients.

Finally, platform trials are prospective, disease-focused, adaptive, RCTs that compare multiple, simultaneous and possibly differently timed interventions against a single, constant control group. This could be of particular interest in cSLE since the reference group treated as per current standard of care might be the most suitable control group for all new medicines to be tested in cSLE and in other rare paediatric rheumatology diseases. Unfortunately, this kind of design has not yet been realised in paediatric rheumatology, possibly because infrastructure support is lacking to make participation in such trials attractive to the pharmaceutical companies.

In conclusion, an open label trial, within the context of a platform registry, with sample size driven by PK purposes, inclusion of PD evaluations, coupled with modelling and simulation, extrapolation of efficacy from adult trial results, followed by long term extension studies with a greater number of patients for safety purposes could potentially be considered an alternative to provide data in support of an indication in cSLE and should be considered by regulators.

Platform based registries could be feasible within the framework of large networks such as PRINTO and PRCSG, as well as the Childhood-Arthritis Research Alliance (CARRA) (55), the UK Juvenile Lupus Registry (56), or GLADEL (57). In the *Report From The Commission To The European Parliament And The Council State of Paediatric Medicines in the EU – 10 years of the EU Paediatric Regulation* (26 October 2017) (58) it is indeed stated in section 4: the last 10 years have seen some considerable progress in the availability of medicines for children in certain therapeutic fields because of the Regulation. Rheumatology or infectious diseases are often referred to as prime examples. The significant surge of new treatments for children with rheumatologic diseases following the completion of PIPs has transformed a sector which was previously neglected. This was facilitated through the involvement of large networks in the planning and conduct of studies.

## Conclusion

Current paediatric development plans for cSLE show overall consistency among themselves and adherence to agreed guidelines. This is needed to allow the generation of interpretable results that enable robust conclusions, underpinning an indication that includes paediatric patients. However, there are recognised obstacles in the conduct of traditionally designed RCTs in cSLE.

Paediatric investigation plans are generally agreed when adult efficacy data are not yet available, but as soon as the product lifecycle progresses, new methods and approaches can offer some advantages over traditional trial design and provide a comparable level of evidence of efficacy in an alternative way.

Extrapolation of adult efficacy data to paediatrics is one possible approach, provided that adult trials produce data that can be used for this purpose and that the degree of residual uncertainty is appropriately quantified for the medicinal product in question. Therefore, such approaches should be explored, planned and thoroughly discussed with regulators. As regulatory experience increases, innovative trial designs such as platform trials may become viable alternatives. Collaboration between regulators, global research networks and increased stakeholder engagement will ensure that progress is monitored and analysed promptly.
